# Supplementary material for: From perception to valence: a pair of interneurons that assign positive valence to sweet sensation in Drosophila
Source: bioRxiv. 2025 Nov 5:2025.10.31.685871. Preprint. [Version 2] doi: 10.1101/2025.10.31.685871 (PMC12636559; doi:10.1101/2025.10.31.685871)
Supplement: Supplement 1 [file media-1.pdf]

Supplementary Figures

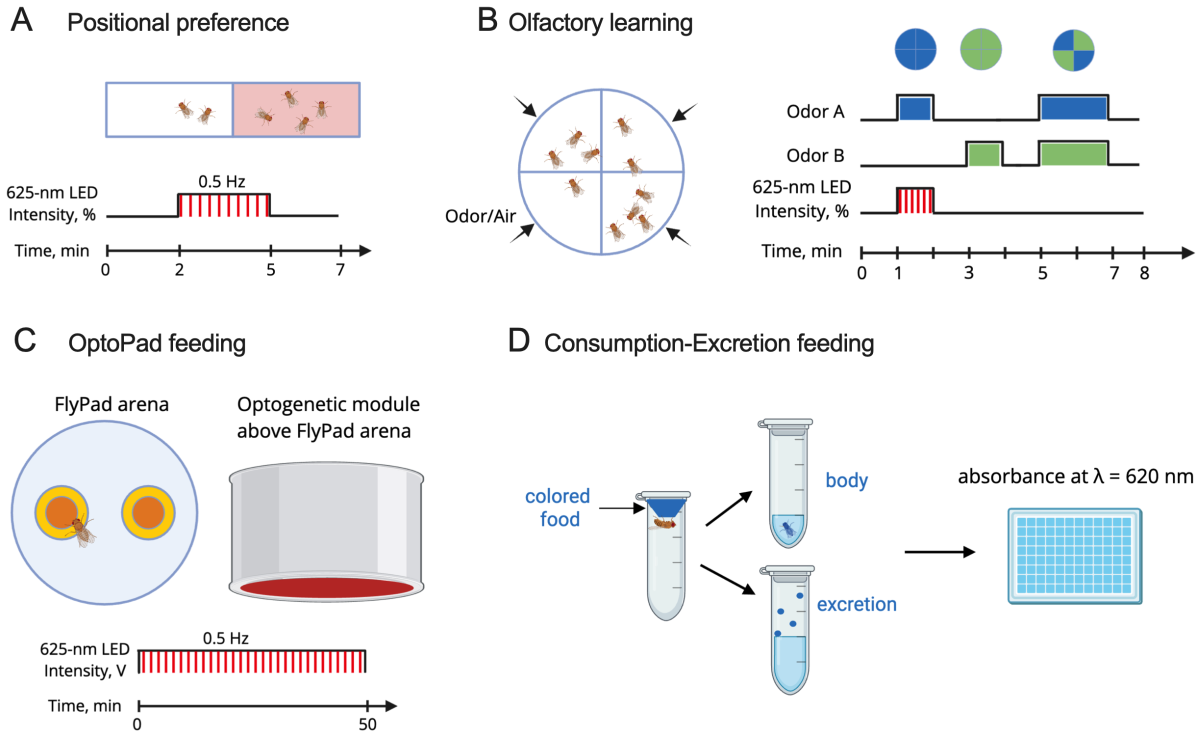

Figure S1. Schematics for behavior assays

A. Positional preference assay: Flies were first loaded into a rectangular arena. Following a 2-minute acclimation period, half of the arena was illuminated with a 625-nm LED for 3 minutes. A subsequent 2-minute recovery period was provided after illumination.

B. Olfactory learning assay: Flies were introduced into a circular arena that was subdivided into four quadrants to enable localized delivery of odors or air. After a 1-minute adaptation period, the arena was illuminated with a 0.5 Hz 625-nm LED while odor A was administered for 1 minute. This was followed by a 1-minute airflow to clear residual odor. Next, odor B was delivered for 1 minute without LED illumination. Finally, after another clearing period, both odors A and B were simultaneously presented from opposite quadrants for 2 minutes to enable a choice.

C. OptoPad feeding assay: This is a FlyPad assay with optogenetic function. A single fly was loaded into a FlyPad arena containing solid food placed at the center of metal rings. Feeding events were recorded when the fly, by contacting the food, completed an electrical circuit. During the open-loop assay, the arena was continuously illuminated by the optogenetic module from above with a 625-nm LED for 50 minutes. Control flies are allowed to feed in the dark, without optogenetic illumination. During the closed-loop assay, the arena was illuminated for 2 seconds by

optogenetic module only when the fly is feeding on one of the probes. The probe associated with activation was alternated for each trial to counterbalance potential intrinsic preference for one probe.

D. Consumption-Excretion (Con-Ex) feeding assay: Flies were maintained on colored food provided in a feeding tube over a 24-hour period. The amount of food consumed was quantified, based on the dye absorbance by recovering and measuring the blue dye from both the homogenized flies and the excretion residues washed from the walls of the feeding tube.

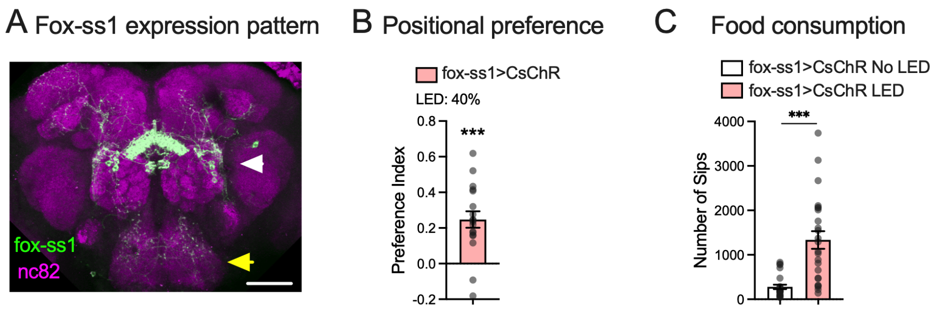

Figure S2. Expression pattern and phenotypes of original fox driver (fox-ss1)

A. Immunostaining of CsChrimson-mVenus driven by fox-ss1 driver lines that labels fox neurons (yellow arrow) and PPM3 dopaminergic neurons (white arrow).

B. Flies preferred the illuminated side in the positional preference assay when their fox neurons were optogenetically activated (fox-ss1>CsChrimson). N = 18. Each replicate contains 6-8 flies. One-sample *t* test (hypothetical value = 0), *** $p<0.001$.

C. Activating fox neurons with fox-ss1 driver increased consumption of food in fed flies. N = 24 flies. *t* test (no LED and LED groups of the same genotype), *** $p<0.001$.

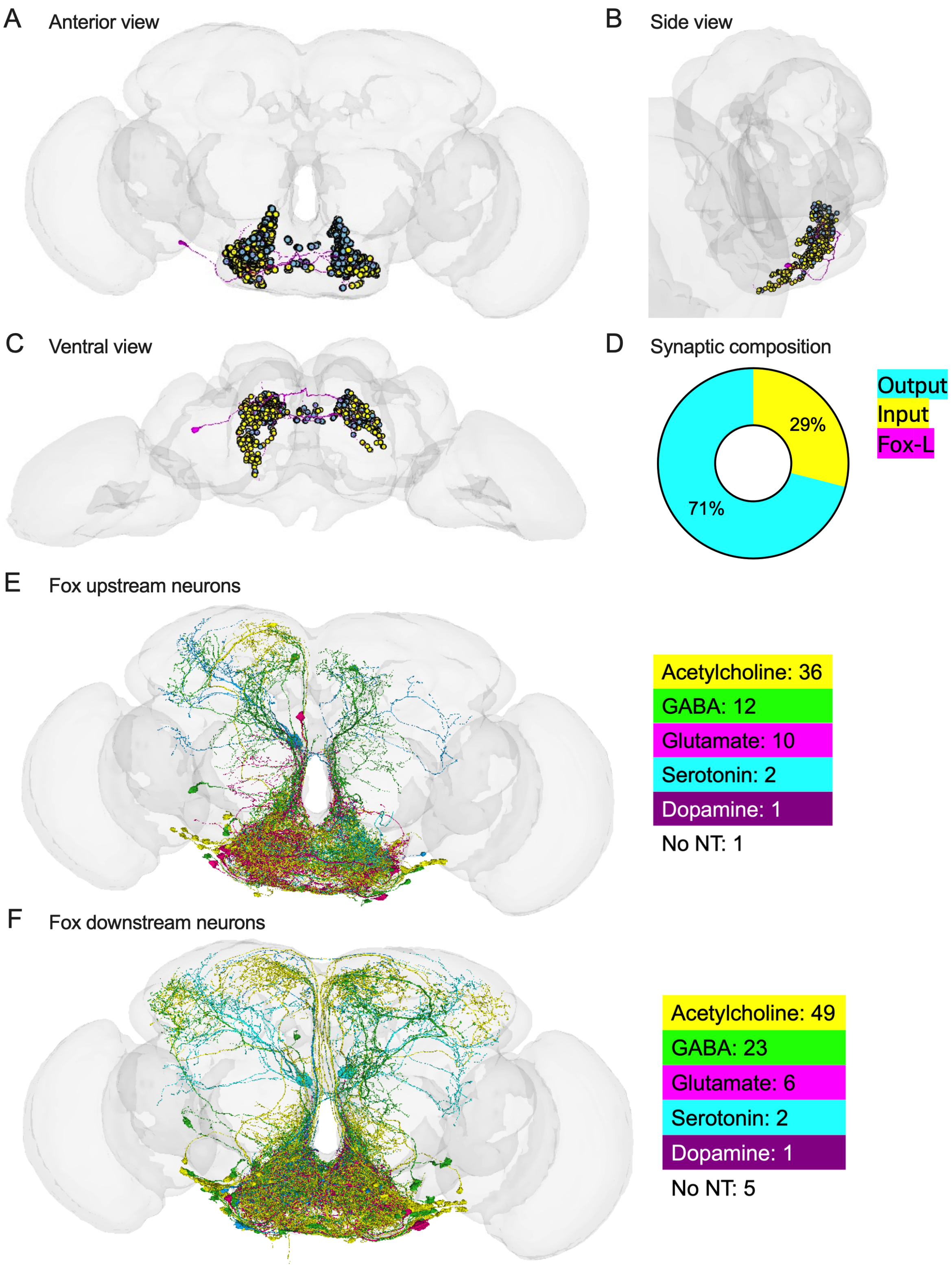

Figure S3. Fox neurons in FAFB.

A-C. Anterior, side, and ventral views of the left-hemisphere fox neuron in FAFB. Yellow dots indicate synaptic inputs; blue dots indicate synaptic outputs.

D. Synaptic composition of the left-hemisphere fox neuron.

E-F. The upstream and downstream synaptic partners of the left-hemisphere fox neuron.

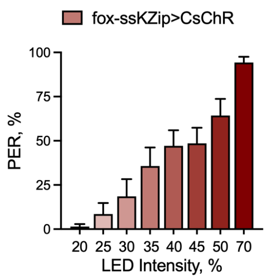

Figure S4. Activating fox neurons induces the proboscis extension reflex

Activating fox neurons (fox-ssKZip>CsChrimson) induced proboscis extension in a dosage-dependent manner. N = 14 flies.

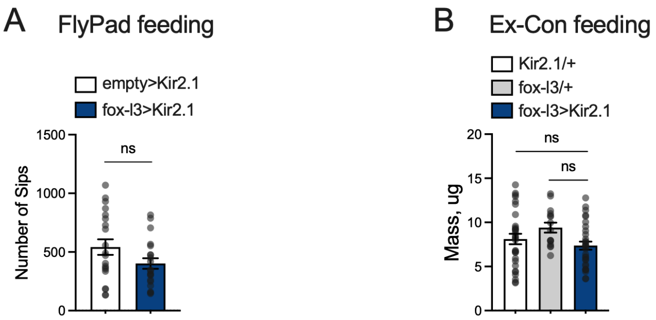

Figure S5. Blocking fox neurons did not affect feeding in fed flies.

A. Constitutively blocking fox neurons with Kir2.1 (fox-l3>Kir2.1) did not affect food consumption in the FlyPad feeding assay compared with genetic control (empty>Kir2.1). N=21 flies for empty>Kir2.1; N=24 flies for fox-l3>Kir2.1. *t* test, ns. p>0.05.

B. Constitutively blocking fox neurons with Kir2.1 (fox-l3>Kir2.1) did not affect 24-hour food consumption compared with genetic controls (Kir2.1/+ and fox-l3/+). N = 30 flies for Kir2.1/+ and fox-l3>Kir2.1; N = 15 flies for fox-l3/+. One-way ANOVA with Tukey's multiple comparison test, ns. P>0.05.

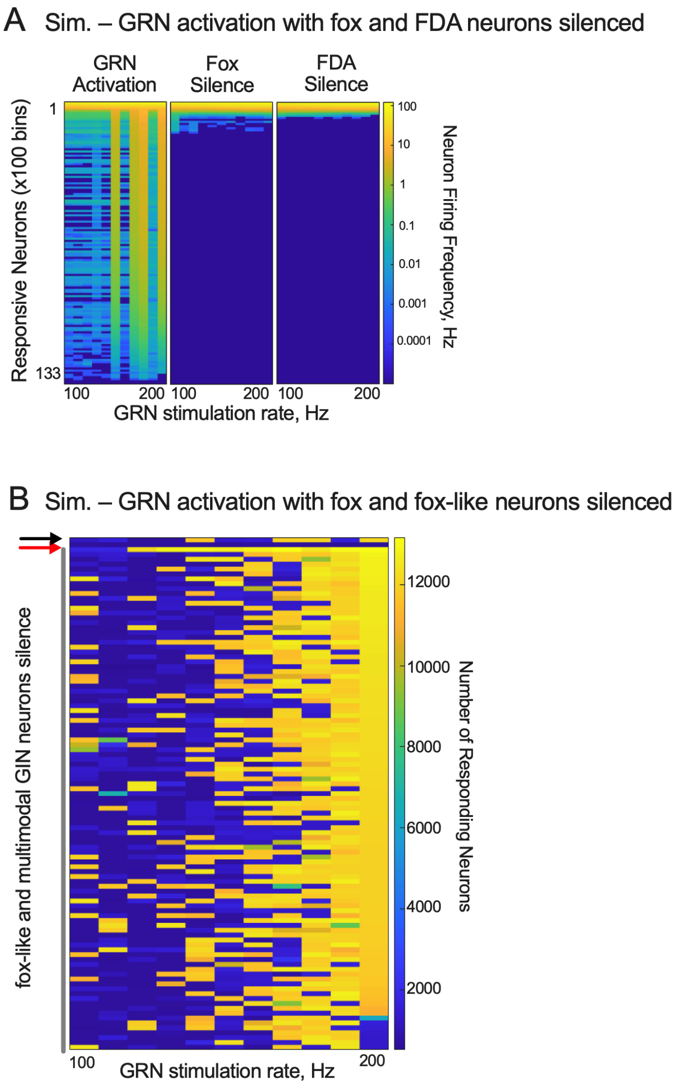

Figure S6. Fox neurons facilitate relaying the positive valence of tastants to the central brain.

A. Simulation of stimulating labellar sweet GRNs without (left panel) and with fox neurons (middle panel) and FDAs (right panel) blocked in computational simulation (model synaptic weight = 0.39), shown as the firing frequency of responsive neurons (averaged 100 neurons/bin).

B. Simulation of blocking the fox-like neurons and multimodal GINs while GRNs were stimulated in computational simulation (model synaptic weight = 0.39). The black arrow indicates the results of GRN activation; the red arrow indicates the results of silencing fox. The grey line indicates the results of silencing fox-like neurons and multimodal GINs (these categories overlap). The heatmap represents the number of responsive neurons in each simulation trial.

Supplementary Tables

Table S1A. Simulated number of responding neurons in the connectome to sugar GRN stimulation during silencing of FDA neuronal pairs or sets.

Silenced neuron(s)	Cell type	20 (Hz)	40 (Hz)	80 (Hz)	100 (Hz)	110 (Hz)	120 (Hz)	130 (Hz)	140 (Hz)	150 (Hz)	160 (Hz)	170 (Hz)	180 (Hz)	190 (Hz)	200 (Hz)
No silencing		644	659	1498	1185	1606	635	658	11720	1321	1431	11946	12043	1616	12169
GNG.SMP.3 / GNG.SMP.8	CB0233	1487	656	613	656	595	620	599	595	602	618	593	574	610	577
GNG.SMP.10 / PRW.SMP.4	CB0546	690	686	630	11056	11972	645	624	637	650	658	598	1294	11825	635
SLP.GNG.3 / SLP.GNG.1	DNp62	645	1266	638	11848	629	638	11328	2467	11674	1314	12022	11954	1691	12053
PRW.SMP.16 / SMP.163	CB0272	697	667	673	633	627	629	12467	11566	1307	623	1455	11943	11269	12201
GNG.79 / GNG.128	CB0337	11740	747	638	649	654	11825	677	1532	11821	12000	12353	11009	12278	11959
GNG.SMP.17 / FLA.SMP.61	CB1514	9789	756	638	1319	630	662	621	1124	11436	12454	1636	12287	11793	1295
FLA.SMP.58 / FLA.SMP.61	CB1514	12149	695	645	1186	676	1181	622	1346	1331	643	1465	11898	12672	11930
GNG.SMP.18 / GNG.SMP.24	CB3199	1266	707	656	625	633	11821	1257	627	1570	654	12239	11454	11947	12326
SMP.1281 / SMP.1493	CB1025	1719	655	657	11669	1260	630	1150	646	1147	1208	12504	11824	11003	12708
GNG.SMP.19 / SMP.2023	CB3470	1355	689	1167	667	627	644	660	12409	1346	1272	11841	11402	11946	12191
SMP.1086 / SMP.1499	CB3573	11386	660	646	1286	627	633	1265	12407	1058	1261	12329	1605	11934	12865
FDA-I		655	1331	673	654	621	12371	620	634	626	617	1407	612	11792	1194
FDA-II		1320	625	606	600	603	632	632	589	597	592	592	594	589	576
FDA-All		634	1355	621	585	599	594	603	599	594	587	614	593	583	592

Table S1B. Simulated number of responding PAM-DANs to sugar GRN stimulation during silencing of FDA neuronal pairs or sets.

Silenced neuron(s)	Cell type	20 (Hz)	40 (Hz)	80 (Hz)	100 (Hz)	110 (Hz)	120 (Hz)	130 (Hz)	140 (Hz)	150 (Hz)	160 (Hz)	170 (Hz)	180 (Hz)	190 (Hz)	200 (Hz)
No silencing		0	0	8	6	8	0	0	200	7	11	201	203	10	201
GNG.SMP.3 / GNG.SMP.8	CB0233	11	0	0	0	0	0	0	0	0	0	0	0	0	0
GNG.SMP.10 / PRW.SMP.4	CB0546	0	0	0	0	202	0	0	200	7	0	7	199	196	205
SLP.GNG.3 / SLP.GNG.1	DNp62	0	7	0	201	0	0	198	10	199	8	202	201	9	202
PRW.SMP.16 / SMP.163	CB0272	0	0	0	0	0	0	201	200	7	0	7	199	196	205
GNG.79 / GNG.128	CB0337	200	0	0	0	0	201	0	8	200	202	203	196	200	200
GNG.SMP.17 / FLA.SMP.61	CB1514	173	0	0	10	0	0	0	7	196	200	11	202	201	7
FLA.SMP.58 / FLA.SMP.61	CB1514	200	0	0	6	0	7	0	7	8	0	11	200	200	202
GNG.SMP.18 / GNG.SMP.24	CB3199	6	0	0	0	0	201	9	0	7	0	201	199	201	200
SMP.1281 / SMP.1493	CB1025	7	0	0	197	9	0	6	0	6	8	203	199	194	205
GNG.SMP.19 / SMP.2023	CB3470	8	0	6	0	0	0	0	200	9	7	199	199	199	202
SMP.1086 / SMP.1499	CB3573	199	0	0	9	0	0	10	200	3	8	200	9	200	202
FDA-I		0	7	0	0	0	200	0	0	0	0	7	0	200	7
FDA-II		10	0	0	0	0	0	0	0	0	0	0	0	0	0
FDA-All		0	9	0	0	0	0	0	0	0	0	0	0	0	0

Table S2. Simulated number of responding PAM-DANs to sugar GRN stimulation with and without silencing Fox, while varying simulation free parameter – synaptic weights (Wsyn)

Condition	Wsyn	10 (Hz)	20 (Hz)	30 (Hz)	40 (Hz)	50 (Hz)	60 (Hz)	70 (Hz)	80 (Hz)	90 (Hz)	100 (Hz)	110 (Hz)	120 (Hz)	130 (Hz)	140 (Hz)	150 (Hz)	160 (Hz)	170 (Hz)	180 (Hz)	190 (Hz)	200 (Hz)
Fox intact	0.37	0	0	0	0	0	0	0	0	0	0	0	0	0	0	0	191	0	5	8	189
Fox intact	0.38	0	0	0	0	0	0	0	0	0	0	0	0	7	8	0	0	8	8	6	196
Fox intact	0.382	0	0	0	0	0	0	0	0	0	0	0	0	0	0	0	8	198	7	6	186
Fox intact	0.386	0	0	0	0	0	0	0	0	0	0	0	0	0	0	0	0	201	201	7	201
Fox intact	0.39	0	0	0	0	0	0	0	8	0	6	8	0	0	200	7	11	201	203	10	201
Fox silenced	0.37	0	0	0	0	0	0	0	0	0	0	0	0	0	0	0	0	0	0	0	0
Fox silenced	0.38	0	0	0	0	0	0	0	0	0	0	0	0	0	0	0	0	0	0	0	0
Fox silenced	0.382	0	0	0	0	0	0	0	0	0	0	0	0	0	0	0	0	0	0	0	0
Fox silenced	0.386	0	6	0	0	0	0	0	0	0	0	0	0	0	0	0	0	0	0	0	0
Fox silenced	0.39	0	0	0	0	0	0	0	0	0	0	0	0	0	0	0	0	0	0	0	0

Table S3A. Simulated number of responding neurons in the connectome to sugar GRN stimulation during silencing of select neuronal pairs or single cells.

Experiment	Silenced neuron(s)	100 (Hz)	110 (Hz)	120 (Hz)	130 (Hz)	140 (Hz)	150 (Hz)	160 (Hz)	170 (Hz)	180 (Hz)	190 (Hz)	200 (Hz)
Control	No silencing	1185	1606	635	658	11720	1321	1431	11946	12043	1616	12169
Fox silence	CB0525 (Fox)	663	603	639	618	617	592	622	622	608	619	646
FDA silence	CB0233	656	595	620	599	595	602	618	593	574	610	577
FDA silence	CB0546	11056	11972	645	624	637	650	658	598	1294	11825	635
FDA silence	DNp62	11848	629	638	11328	2467	11674	1314	12022	11954	1691	12053
FDA silence	CB0272	633	627	629	12467	11566	1307	623	1455	11943	11269	12201
FDA silence	CB0337	649	654	11825	677	1532	11821	12000	12353	11009	12278	11959
FDA silence	CB1514 (1)	1319	630	662	621	1124	11436	12454	1636	12287	11793	1295
FDA silence	CB1514 (2)	1186	676	1181	622	1346	1331	643	1465	11898	12672	11930
FDA silence	CB3199	625	633	11821	1257	627	1570	654	12239	11454	11947	12326
FDA silence	CB1025	11669	1260	630	1150	646	1147	1208	12504	11824	11003	12708
FDA silence	CB3470	667	627	644	660	12409	1346	1272	11841	11402	11946	12191
FDA silence	CB3573	1286	627	633	1265	12407	1058	1261	12329	1605	11934	12865
FDA silence	FDA-I	654	621	12371	620	634	626	617	1407	612	11792	1194
FDA silence	FDA-II	617	603	586	632	613	585	598	601	571	589	582
FDA silence	FDA-All	585	599	594	603	599	594	587	614	593	583	592
Foxlike silence	CB0152	617	633	626	10674	1208	1087	1098	9903	12002	12377	12054
Foxlike silence	CB0117	1287	1240	625	619	622	1126	11744	12428	11839	12052	11677
Foxlike silence	CB3793	644	1524	620	624	631	1354	1263	11687	12460	11911	1359
Foxlike silence	CB0170	635	639	12470	1329	1431	1429	11588	11929	1276	1263	12070
Foxlike silence	CB0251	620	632	629	633	1239	625	1256	642	11935	12389	12093
Foxlike silence	CB0616	9462	1294	635	1313	11741	1246	1287	1185	11957	12319	12242
Foxlike silence	CB0560	11112	645	632	635	627	1191	10776	10583	11042	12604	12370
Foxlike silence	CB0553	528	552	521	1218	549	11628	1572	1290	12349	12065	12415
Foxlike silence	CB0880	675	635	1472	628	11587	1188	1074	11523	12458	11856	12428

Foxlike silence	CB0848	1232	1335	641	1296	11989	630	12473	11659	11800	11981	12209
Foxlike silence	CB0421	617	629	12198	665	639	1243	11640	10218	12338	11883	12495
Foxlike silence	CB0247	1757	1779	12271	12831	12859	12690	12899	13125	12537	13028	13170
Foxlike silence	CB0617	12446	1632	671	1318	12533	11661	785	947	12372	12119	12700
Foxlike silence	CB0473	644	623	644	11445	607	11467	1384	12524	11871	12008	12323
Foxlike silence	CB0331	625	643	11710	1334	1079	1275	12427	11982	12153	12686	12005
Foxlike silence	CB0287	1279	1502	621	643	11852	1365	12317	1106	11807	12694	11897
Foxlike silence	CB0791	662	657	649	669	823	11692	11759	11761	11844	12193	12275
Foxlike silence	CB0768	11762	1374	1281	633	1671	12388	11789	11705	12146	11503	12277
Foxlike silence	CB0437	1348	621	657	630	666	1225	1251	1088	1302	12073	12066
Foxlike silence	CB0186	616	1107	12448	604	11829	1273	1718	1165	11254	12394	12320
Foxlike silence	CB0502	623	621	11704	1635	636	11859	12003	12269	11783	11956	11994
Foxlike silence	CB2820	1324	660	1311	631	12099	12426	9624	1256	12633	11839	12031
Foxlike silence	CB0489	626	1312	627	639	12428	12419	1257	11832	9409	12195	12764
Foxlike silence	CB0900	1357	622	633	629	675	636	11622	1252	12640	12414	12733
Foxlike silence	CB0737	651	11746	665	11418	11852	640	1082	7796	1174	11877	12002
Foxlike silence	CB0923	656	640	678	624	12472	1210	11840	12549	12089	12028	12007
Foxlike silence	CB0479	1512	638	635	950	1131	11750	1252	11924	11963	12581	11527
Foxlike silence	CB0587	12503	619	1589	632	11840	1351	12317	12252	1666	11810	11992
Foxlike silence	CB0797	628	638	644	691	631	12649	1101	11466	12033	11564	12696
Foxlike silence	CB0707	663	11697	623	627	666	632	12006	12509	12540	12633	11939
Foxlike silence	CB0137	12525	663	1141	636	672	1214	633	11859	1316	12497	1343
Foxlike silence	CB0014	618	640	656	626	1807	12356	1096	1376	12541	12457	1345
Foxlike silence	CB0552	11913	628	636	11910	673	1305	11746	12375	11880	11996	12013
Foxlike silence	CB0238	11379	646	1145	643	624	10810	1403	12543	1588	11878	12355
Foxlike silence	CB1779	681	630	615	10793	647	1318	1391	1419	12431	12214	11762
Foxlike silence	CB2115	1265	1395	629	634	1473	1234	11995	1223	12381	11821	12327
Foxlike silence	CB1475	627	626	621	659	622	645	12192	9208	12132	1404	11441
Foxlike silence	CB0917	624	620	631	1317	655	1226	12027	11705	1406	12751	12546
Foxlike silence	CB2385	623	641	1107	619	1122	11993	633	11662	12535	12085	11170
Foxlike silence	CB0795	672	1553	11775	1293	1062	1382	1151	10555	12119	12061	12589
Foxlike silence	CB0468	628	637	633	626	1114	11659	11317	12307	12236	11901	12346
Foxlike silence	CB0434	615	637	12041	1158	11750	1221	12090	10937	12034	1499	1505

Foxlike silence	CB0893	713	625	627	1241	11912	11984	1533	1289	1385	11943	12584
Foxlike silence	CB2039	642	1360	626	642	1164	11833	642	1656	10847	12372	12201
Foxlike silence	CB0515	635	12323	657	12526	656	1324	1415	1349	11950	8720	11927
Foxlike silence	CB0908	11552	657	12554	633	640	1452	12202	11588	11900	1293	12123
Foxlike silence	CB0108	1380	648	631	633	663	10293	11632	1238	11910	1663	11811
Foxlike silence	CB2403	658	627	12552	1782	665	1423	1352	11103	12575	11554	12150
Foxlike silence	CB0910	643	604	1149	1256	1233	11795	1324	12544	12183	11889	12492
Foxlike silence	CB0445	625	1254	1636	11695	11657	11769	11925	12688	11960	12714	12479
Foxlike silence	CB0604	645	12333	1363	1473	12218	931	1091	1549	11868	11024	11876
Foxlike silence	CB3615	710	619	1322	633	1353	12225	12599	12400	11888	12318	12110
Foxlike silence	CB0277	624	655	11873	657	11453	1362	10697	12468	11786	12674	1581
Foxlike silence	CB0360	10145	2132	10876	661	630	11511	1499	11044	11859	12244	12246
Foxlike silence	CB1563	619	629	1284	645	1290	12259	1077	11571	12512	11856	12268
Foxlike silence	CB0278	11028	1288	1349	649	1184	716	1246	11919	12581	11984	12566
Foxlike silence	CB0885	649	6804	1213	1147	627	11825	1179	657	1118	12524	12119
Foxlike silence	CB0549	634	629	668	637	668	1344	1566	1231	1350	12479	11961
Foxlike silence	Unknown cell type - GNG.706, GNG.656	644	628	640	643	11648	642	1320	1316	12386	12121	12014
Foxlike silence	CB0884	657	11801	634	622	1276	11990	12271	10431	10511	11746	11924
Foxlike silence	CB0507	653	11694	1430	637	1360	625	1103	11865	11708	12075	12097
Foxlike silence	CB0542	614	616	615	616	604	1202	609	1085	12070	11641	12113
Foxlike silence	CB0811	649	643	1355	624	1371	1177	11893	12069	1405	12699	12095
Foxlike silence	CB3714	11957	636	609	1107	1129	11627	11749	12500	1252	11833	12454
Foxlike silence	CB0879	661	644	661	627	647	1322	12520	11723	12092	12026	11910
Foxlike silence	CB0177	631	618	1190	662	10946	1006	680	2267	11885	12445	11819
Foxlike silence	CB0106	1149	658	1217	651	662	1160	669	11850	12589	12070	12538
Foxlike silence	CB0581	612	1344	623	11745	1628	11717	12038	1609	12490	12012	12746
Foxlike silence	CB0038	654	618	661	1139	650	11635	1266	11853	12043	11716	12412
Foxlike silence	CB0493	1232	614	657	635	633	620	11919	1477	12016	12440	12758
Foxlike silence	CB0803	1253	626	646	653	665	11116	1281	1219	11942	12063	12000
Foxlike silence	CB0765	11113	610	637	649	11736	1116	1438	11421	11282	12045	11778
Foxlike silence	CB3812	1141	611	638	676	628	11951	11564	1604	9556	12812	12154
Foxlike silence	CB0775	11533	646	623	638	11316	1558	1368	11608	12537	11841	12613
Foxlike silence	CB0239	12031	630	1163	1571	11604	11770	11780	1277	9637	12065	12016
Foxlike silence	CB0523	622	1399	12283	1512	644	1511	1375	1574	12459	11798	12063

Foxlike silence	CB2606	614	631	635	1216	1141	1236	9954	1432	12044	11838	12643
Foxlike silence	CB0855	12111	1240	666	11604	11245	650	1168	11741	1350	11148	11950
Foxlike silence	CB0521	11554	8791	627	644	1376	637	11743	12129	11768	11973	12256
Foxlike silence	CB0216	1440	630	644	626	1348	639	12627	11501	12496	1533	12440
Foxlike silence	CB0190	615	1330	628	641	12310	12142	636	1224	12033	11951	11898
Foxlike silence	CB0864	11850	1272	666	1223	626	636	1378	11963	11958	12250	12007
Foxlike silence	CB0457	1397	623	630	674	1331	1338	1163	11804	12067	12665	12230
Foxlike silence	CB0756	1269	1430	1211	625	1214	1147	11403	11932	11754	12293	12850
Foxlike silence	CB0844	626	634	635	1235	635	639	12029	1381	1630	11681	12491
Foxlike silence	CB1093	624	612	649	812	635	636	642	11831	11785	12030	1403
Foxlike silence	CB0759	11533	650	629	655	11929	12191	11242	1495	12659	11572	11862
Foxlike silence	CB0292	632	626	668	664	11749	666	11540	11750	1381	12821	11664
Foxlike silence	CB0731	666	643	631	628	11689	1194	1414	1295	10876	12345	12063
Foxlike silence	CB2606	624	658	636	632	11483	1579	11549	12551	12290	12296	11801
Foxlike silence	CB1579	663	1036	626	11522	11866	1270	1345	648	1478	11670	11787
Foxlike silence	CB0921	12340	619	627	626	630	627	607	12013	1500	11891	12308
Foxlike silence	CB0799	655	1216	651	1085	11007	1126	644	11980	12031	1235	6221
Foxlike silence	CB0823	1313	661	1421	1433	647	1186	1354	1308	1584	11769	12316
Foxlike silence	CB2820	630	1214	11744	11771	1261	1041	1332	12049	12116	12463	12274
Foxlike silence	CB2014	1365	1131	1226	1427	1237	12484	11819	12618	12140	11863	12184
Foxlike silence	CB1470	1266	611	632	634	10937	11966	1231	11562	11937	12274	12161
Foxlike silence	CB0731	628	668	1067	12331	1303	627	643	12043	1321	12445	12305
Foxlike silence	CB0988	624	1111	634	697	633	11579	1409	1374	1272	12264	11131
Foxlike silence	CB1470	1207	623	637	643	1311	1337	11677	11646	1126	11409	12027
Foxlike silence	CB0400	11957	668	632	645	1478	11342	1351	11750	11914	1437	12420
Foxlike silence	Unknown cell type - GNG.758	12362	638	666	1326	623	631	11894	1325	11797	11961	12579
Foxlike silence	CB0811	1291	620	621	11645	1309	1703	12092	11728	10553	11583	11976

Table S3B. Simulated number of responding PAM-DANs to sugar GRN stimulation during silencing of select neuronal pairs or single cells.

Experiment	Silenced neuron(s)	100 (Hz)	110 (Hz)	120 (Hz)	130 (Hz)	140 (Hz)	150 (Hz)	160 (Hz)	170 (Hz)	180 (Hz)	190 (Hz)	200 (Hz)
Control	No silencing	6	8	0	0	200	7	11	201	203	10	201
Fox silence	CB0525 (Fox)	0	0	0	0	0	0	0	0	0	0	0
FDA silence	CB0233	0	0	0	0	0	0	0	0	0	0	0
FDA silence	CB0546	0	202	0	0	200	7	0	7	199	196	205
FDA silence	DNp62	201	0	0	198	10	199	8	202	201	9	202
FDA silence	CB0272	0	0	0	201	200	7	0	7	199	196	205
FDA silence	CB0337	0	0	201	0	8	200	202	203	196	200	200
FDA silence	CB1514 (1)	10	0	0	0	7	196	200	11	202	201	7
FDA silence	CB1514 (2)	6	0	7	0	7	8	0	11	200	200	202
FDA silence	CB3199	0	0	201	9	0	7	0	201	199	201	200
FDA silence	CB1025	197	9	0	6	0	6	8	203	199	194	205
FDA silence	CB3470	0	0	0	0	200	9	7	199	199	199	202
FDA silence	CB3573	9	0	0	10	200	3	8	200	9	200	202
FDA silence	FDA-I	0	0	200	0	0	0	0	7	0	200	7
FDA silence	FDA-II	0	0	0	0	0	0	0	0	0	0	0
FDA silence	FDA-All	0	0	0	0	0	0	0	0	0	0	0
Foxlike silence	CB0152	0	0	0	192	7	6	5	170	201	201	201
Foxlike silence	CB0117	10	8	0	0	0	7	201	200	201	200	201
Foxlike silence	CB3793	0	10	0	0	0	9	9	202	202	201	10
Foxlike silence	CB0170	0	0	203	10	9	9	200	202	8	7	200
Foxlike silence	CB0251	0	0	0	0	6	0	8	0	200	201	201
Foxlike silence	CB0616	165	8	0	7	200	7	7	7	201	202	200
Foxlike silence	CB0560	196	0	0	0	0	6	195	185	195	201	200
Foxlike silence	CB0553	0	0	0	7	0	199	9	8	202	202	203
Foxlike silence	CB0880	0	0	8	0	202	7	5	199	200	200	200
Foxlike silence	CB0848	7	8	0	7	203	0	203	200	201	201	204

Foxlike silence	CB0421	0	0	201	0	0	7	199	190	199	201	200
Foxlike silence	CB0247	11	11	202	205	204	204	203	204	205	205	207
Foxlike silence	CB0617	201	8	0	7	202	200	0	3	201	200	204
Foxlike silence	CB0473	0	0	0	197	0	199	10	200	201	203	203
Foxlike silence	CB0331	0	0	200	7	5	8	205	202	201	202	201
Foxlike silence	CB0287	7	10	0	0	201	10	203	5	200	202	200
Foxlike silence	CB0791	0	0	0	0	0	200	200	201	200	201	204
Foxlike silence	CB0768	201	8	7	0	8	203	200	200	201	200	203
Foxlike silence	CB0437	10	0	0	0	0	8	7	5	8	201	203
Foxlike silence	CB0186	0	5	201	0	202	7	7	6	199	200	203
Foxlike silence	CB0502	0	0	199	8	0	201	204	200	200	200	203
Foxlike silence	CB2820	9	0	8	0	201	201	170	7	203	202	200
Foxlike silence	CB0489	0	7	0	0	201	201	7	200	166	199	202
Foxlike silence	CB0900	7	0	0	0	0	0	200	6	202	199	203
Foxlike silence	CB0737	0	199	0	199	202	0	5	91	7	202	200
Foxlike silence	CB0923	0	0	0	0	201	7	201	202	203	202	201
Foxlike silence	CB0479	10	0	0	3	6	200	7	201	201	201	200
Foxlike silence	CB0587	200	0	8	0	202	9	201	204	10	200	199
Foxlike silence	CB0797	0	0	0	0	0	203	6	198	203	198	203
Foxlike silence	CB0707	0	202	0	0	0	0	202	203	204	202	200
Foxlike silence	CB0137	200	0	7	0	0	7	0	201	7	203	7
Foxlike silence	CB0014	0	0	0	0	11	199	5	10	203	201	7
Foxlike silence	CB0552	201	0	0	202	0	10	202	199	201	203	203
Foxlike silence	CB0238	199	0	6	0	0	195	9	203	10	201	202
Foxlike silence	CB1779	0	0	0	192	0	8	8	9	201	201	202
Foxlike silence	CB2115	7	10	0	0	7	7	203	7	201	200	201
Foxlike silence	CB1475	0	0	0	0	0	0	201	144	201	11	196
Foxlike silence	CB0917	0	0	0	7	0	7	200	200	9	202	202
Foxlike silence	CB2385	0	0	6	0	7	202	0	198	203	202	197
Foxlike silence	CB0795	0	7	201	8	6	10	6	185	202	201	202
Foxlike silence	CB0468	0	0	0	0	6	200	200	200	200	201	201
Foxlike silence	CB0434	0	0	199	7	201	7	201	193	202	8	8
Foxlike silence	CB0893	0	0	0	7	203	201	10	9	12	202	202

Foxlike silence	CB2039	0	7	0	0	7	203	0	9	193	200	202
Foxlike silence	CB0515	0	202	0	201	0	8	8	8	201	122	200
Foxlike silence	CB0908	200	0	204	0	0	8	200	201	201	7	203
Foxlike silence	CB0108	11	0	0	0	0	184	201	7	202	9	200
Foxlike silence	CB2403	0	0	200	8	0	10	12	196	202	199	203
Foxlike silence	CB0910	0	0	6	7	7	199	10	202	202	202	203
Foxlike silence	CB0445	0	8	10	199	200	200	201	203	201	203	202
Foxlike silence	CB0604	0	200	8	8	201	3	5	7	200	199	200
Foxlike silence	CB3615	0	0	9	0	7	200	202	202	200	203	202
Foxlike silence	CB0277	0	0	199	0	200	10	188	200	200	201	7
Foxlike silence	CB0360	182	9	190	0	0	199	9	195	201	201	201
Foxlike silence	CB1563	0	0	9	0	7	201	6	199	202	200	202
Foxlike silence	CB0278	196	7	7	0	7	0	7	198	203	202	202
Foxlike silence	CB0885	0	63	7	6	0	199	7	0	6	203	202
Foxlike silence	CB0549	0	0	0	0	0	7	10	6	9	202	203
Foxlike silence	Unknown cell type - GNG.706, GNG.656	0	0	0	0	200	0	7	11	201	202	201
Foxlike silence	CB0884	0	199	0	0	8	200	199	188	187	200	201
Foxlike silence	CB0507	0	201	6	0	9	0	7	202	200	200	200
Foxlike silence	CB0542	0	0	0	0	0	7	0	6	203	200	202
Foxlike silence	CB0811	0	0	10	0	9	7	201	201	8	202	201
Foxlike silence	CB3714	200	0	0	5	6	198	201	202	7	200	203
Foxlike silence	CB0879	0	0	0	0	0	7	202	200	203	201	201
Foxlike silence	CB0177	0	0	6	0	196	5	0	9	200	202	201
Foxlike silence	CB0106	6	0	6	0	0	7	0	201	203	200	202
Foxlike silence	CB0581	0	7	0	202	10	200	201	10	203	200	200
Foxlike silence	CB0038	0	0	0	6	0	200	7	199	202	200	203
Foxlike silence	CB0493	5	0	0	0	0	0	202	8	201	202	203
Foxlike silence	CB0803	7	0	0	0	0	195	7	7	202	203	201
Foxlike silence	CB0765	194	0	0	0	201	6	8	198	196	200	199
Foxlike silence	CB3812	4	0	0	0	0	201	198	8	160	204	203
Foxlike silence	CB0775	199	0	0	0	199	10	8	199	201	199	202
Foxlike silence	CB0239	200	0	6	10	200	201	200	7	170	200	202
Foxlike silence	CB0523	0	7	201	10	0	8	9	7	202	199	202
Foxlike silence	CB2606	0	0	0	7	6	7	177	9	202	200	200

Foxlike silence	CB0855	202	7	0	200	198	0	6	200	10	195	200
Foxlike silence	CB0521	202	124	0	0	9	0	201	201	201	203	202
Foxlike silence	CB0216	8	0	0	0	7	0	204	201	201	9	204
Foxlike silence	CB0190	0	7	0	0	202	200	0	7	200	202	201
Foxlike silence	CB0864	199	8	0	7	0	0	7	201	202	203	200
Foxlike silence	CB0457	7	0	0	0	11	7	5	201	200	202	201
Foxlike silence	CB0756	7	10	6	0	7	7	197	201	202	202	202
Foxlike silence	CB0844	0	0	0	8	0	0	200	10	12	198	202
Foxlike silence	CB1093	0	0	0	0	0	0	0	201	200	202	9
Foxlike silence	CB0759	198	0	0	0	202	203	199	10	202	200	200
Foxlike silence	CB0292	0	0	0	0	201	0	199	200	8	203	199
Foxlike silence	CB0731	0	0	0	0	200	7	11	8	196	203	201
Foxlike silence	CB2606	0	0	0	0	201	8	201	201	202	203	201
Foxlike silence	CB1579	0	6	0	199	201	8	10	0	7	200	200
Foxlike silence	CB0921	201	0	0	0	0	0	0	203	9	201	203
Foxlike silence	CB0799	0	9	0	5	194	6	0	201	201	7	57
Foxlike silence	CB0823	10	0	10	9	0	6	9	9	10	201	205
Foxlike silence	CB2820	0	7	199	201	7	6	8	201	200	202	202
Foxlike silence	CB2014	8	6	9	7	9	202	200	202	202	202	201
Foxlike silence	CB1470	9	0	0	0	194	200	7	200	201	205	201
Foxlike silence	CB0731	0	0	6	201	10	0	0	201	10	201	203
Foxlike silence	CB0988	0	6	0	0	0	196	8	8	7	202	194
Foxlike silence	CB1470	9	0	0	0	8	7	199	199	6	198	202
Foxlike silence	CB0400	199	0	0	0	8	199	7	201	199	8	201
Foxlike silence	Unknown cell type - GNG.758	201	0	0	8	0	0	201	8	202	200	201
Foxlike silence	CB0811	8	0	0	198	7	9	202	200	191	200	201
